# Assessing Osteoporosis Knowledge and Beliefs Among Adults Living in Saudi Arabia: A Cross-Sectional Study

**DOI:** 10.7759/cureus.53466

**Published:** 2024-02-02

**Authors:** Anas M Fallatah, Alaa M Fallatah, Abdulaziz Hariri, Faisal Alshadadi, Wid S Al-Abbadi, Mohammed S Alsaad, Bayan Ghalimah, Amre S Hamdi

**Affiliations:** 1 Internal Medicine, King Faisal Specialist Hospital and Research Centre, Riyadh, SAU; 2 College of Medicine, King Abdulaziz University Faculty of Medicine, Jeddah, SAU; 3 Orthopedics and Trauma Surgery, Hôpitaux Universitaires de Marseille, Marseille, FRA; 4 General Surgery, King Abdulaziz University Hospital, Jeddah, SAU; 5 Obstetrics and Gynecology, Dr. Soliman Fakeeh Hospital, Jeddah, SAU; 6 Orthopedic Surgery, International Medical Center Hospital, Jeddah, SAU; 7 Orthopedic Surgery, King Abdulaziz University Faculty of Medicine, Jeddah, SAU

**Keywords:** saudi arabia, awareness, beliefs, knowledge, osteoporosis

## Abstract

Background: Knowledge and beliefs about osteoporosis have been considered one of the vital parts of early prevention against it.

Objectives: This study aimed to evaluate knowledge and beliefs toward osteoporosis using the Osteoporosis Knowledge Assessment Tool (OKAT) and Osteoporosis Health Belief Scale (OHBS) questionnaires among the public in Jeddah, Saudi Arabia.

Methods: This cross-sectional study was conducted from March 2019 to April 2019 among adults aged 15 years and above. A validated questionnaire was allocated electronically to the participants through social platforms (such as Twitterand WhatsApp) using a convenience sampling technique.

Results: A total of 754 participants completed the questionnaire. The majority were females 481 (63.8%). A total of 34 (4.1%) have not heard about osteoporosis before. Respondents scored a total mean of 7.92±3.0for the OKAT questionnaire and a mean score of 126.74±22.38for the OHBS questionnaire. These two scores were significantly associated with age groups and gender (P < 0.05).

Conclusion: Although there is a relative increase in the knowledge of our sample, the belief towardosteoporosis is evidently lower. Therefore, implementing educational programs that tackle belief perception and other preventive measures such as healthy eating habits, physical activities, and educational materials are needed in the future.

## Introduction

Osteoporosis is defined as a skeletal disorder characterized by compromised bone strength, predisposing a person to an increased risk of fracture. Bone strength includes two main features, bone density and bone quality [1]. Osteoporosis has a significant impact on the affected person’s life, as well as their families and the population. It can lead to significant physical, psychological, and financial consequences after the first or second fracture. Otherwise, it is considered as a “silent” disorder until it causes fractures.

It is estimated that over 200 million people worldwide have osteoporosis, and its prevalence is continuing to rise as the elderly population is increasing [2]. Multiple studies have shown that osteoporosis is far more prevalent in Saudi Arabia than in Western countries, which could be explained by the widespread vitamin D deficiency among the Saudi population overall, particularly in women, children, and adolescents [3].

Osteoporosis cannot be cured; however, it can be prevented by several lifestyle habits, such as an increased level of physical activity and adequate consumption of dietary vitamin D and calcium [4,5]. Optimization of peak bone mass in earlier years is thought to play a major role in osteoporosis prevention later in life [6]. It was recently reported that 78% of the Saudi population knew about osteoporosis; females are more knowledgeable than males [7]. Notable evidence suggests that knowledge about osteoporosis contributes to its preventive behavior; however, this is not a sharply defined association [8-11].

Many young adults do not practice healthy preventative lifestyle habits as they do not identify themselves as at risk of developing osteoporosis in the future. In addition, as mentioned previously, osteoporosis is known as a “silent” disorder since its consequences are not immediately evident, which further allows young adults to believe they are highly unsusceptible to developing the disorder in the future. Furthermore, it may be perceived as a disorder that only affects older women. Recent studies have proved that men are also susceptible to osteoporosis. Despite age and gender, insufficient consideration was paid to the seriousness of osteoporosis and the associated health motivation [6].

The osteoporosis dilemma in Saudi Arabia has been disregarded until recently. Numerous studies showed several misperceptions about osteoporosis and its risk factors [12]. The assessment of knowledge and beliefs about osteoporosis among the Jeddah population would help correct common misperceptions, raise awareness about the seriousness of the matter, and improve the population’s preventive lifestyle practices.

## Materials and methods

This cross-sectional study was done between March 2019 and April 2019 in Jeddah, Saudi Arabia. Our sample consisted of Jeddah residents, Saudi and non-Saudi males and females ages 15 and above, on social network platforms, such as WhatsAppMessenger and Twitter applications. Ethical approval to conduct the study was obtained from the King Abdulaziz University Hospital Institutional Review Board (657-23).

A new Arabic questionnaire was developed to evaluate knowledge, attitudes, and beliefs among the public towardosteoporosis. It consisted of three main parts. The first part addressed the sociodemographic information of the population of interest, including the city of residence, gender, age, height, weight, marital status, education, health insurance status, and monthly income.

The second part assessed the knowledge and awareness towardosteoporosis using the Osteoporosis Knowledge Assessment Tool (OKAT) questionnaire. The Arabic version is shown in Tables 8, 9 (see Appendices). It consisted of four areas regarding basic knowledge of osteoporosis, which covered risk factors, preventive methods, recognition of the disease, and treatment availability. It includes a set of 20 questions answered by correct, incorrect, or I do not know. The total score ranges from 0 to 20; having one score for correct, and 0 for incorrect or I do not know.

The third part assessed the beliefs about osteoporosis using the Osteoporosis Health Belief Scale (OHBS) survey. Its Arabic version is shown in Tables 10, 11 (see Appendices). It is composed of seven categories; each category consists of six items making it 42 questions in total. These categories addressed the realization of vulnerability to osteoporosis, appreciation of the severity of the disease, perception of barriers to calcium intake, exercise, and health motivation. The questions were answered on a Likert scale of 5 points, which are strongly agree, agree, disagree, strongly disagree, and neutral.

To decide whether the study objectives were accomplished by the questionnaire, the questions were reviewed by specialized clinicians and medical education staff in the College of Medicine at King Abdulaziz University for face validity. Accordingly, the questions were adjusted, and the questionnaire was also tested by doing a small pilot survey allocated to 23 respondents, of the same targeted population, to ensure that the questionnaire is clear and understandable. Thus, few changes were made regarding the questions’ phrases and numbers before distributing the questionnaire to the whole sample. For the reliability of the responses to the questionnaire, we used the method of test and re-test as it is more appropriate to the study questionnaire, especially the second and third parts of the questionnaire that evaluated OKAT and OHBS. The questionnaire was distributed to 23 respondents, and their answers were recorded. After two weeks, the same respondents were asked to complete the same questionnaire. The coefficient of correlation between the two sets of responses was calculated to test the consistency and repeatability of answers. The value of the coefficient was determined to be 0.794 and 0.894, respectively, for OKAT and OHBS, which indicated a high degree of correlation and consistency between the responses at the two different time points. The questionnaire was electronically distributed through social network platforms (e.g., WhatsAppMessenger and Twitter) for those who live in Jeddah [13,14].

A total of 1003 participated in the study. Those who responded outside Jeddah (n=241) were removed. Overall, 754 participants were included for further analysis. Data management and analysis were carried out using the StatisticalPackage for the Social Sciences (IBMSPSSStatisticsforWindows,IBMCorp., Version 26.0, Armonk,NY)for Mac software. Three types of analysis were carried out, univariate descriptive statistics, in terms of frequencies and percentages for categorical variables. Bivariate statistics such as Chi-square and one-way analysis of variance (ANOVA) to assess the association between the demographics and different parts of the questionnaire.

## Results

Our aim was to assess the knowledge and beliefs about osteoporosis among the population at risk in Jeddah City, Saudi Arabia. A total of 754 out of 1004 responses were included in the analysis after applying both the inclusionand exclusion criteria, and the elimination of incomplete responses. A total of 723 (95.9%) of which had heard before about osteoporosis, 481 (63.8%) were females, 467 (61.9%) were in the age group between 15 and 29 years old, and 432 (57.3%) had an estimated monthly salary of less than five thousand Saudi Riyals. Demographic characteristics are summarized in Table 1.

**Table 1:** Demographic characteristics of the respondents n = Total data, SR = Saudi Riyals,Cms = Centimeters(Means ± Standard Deviation),P: Partial correlation coefficients P*-*value was carried out using bivariate statistics such as Chi-square and one-way analysis of variance (ANOVA).

Categories	Males n=273	Females n=481	Total n=754	P-values
Weight in Kgs:	(80.9 ± 20.7)	(64.7 ± 15.8)	754	P≤ 0.000
Height in Cms:	(172.5 ± 7.6)	(160.1 ± 6.1)	754	P≤ 0.000
Body Mass Index Values	(27.2 ± 6.9)	(25.2 ± 6.0)	754	P≤ 0.000
Nationality:				
Saudi	256	456	712	P≤ 0.621
Non-Saudi	17	25	42	
Educational Levels:				
Elementary or less	0	0	0	P≤ 0.015
Intermediate/High Schools	108	141	249	
College Degree (Bachelor)	142	289	431	
Post Graduate/Doctoral	23	51	74	
Estimated Monthly Salary:				
Less than 5000 SR	177	255	432	P≤ 0.000
5000 to 10000 SR	36	84	120	
10000 to 20000 SR	23	96	119	
More than 20000 SR	37	46	83	
Age Groups:				
15 to 29 Years old	204	263	467	P≤ 0.000
30 to 44 Years old	37	139	176	
45 to 60 Years old	29	76	105	
More than 60 Years old	3	3	6	
Most of Medical Expenses are Paid Through:				
Health Insurance	124	197	321	P≤ 0.263
Government/Military (Free)	65	129	194	
Personal Account (not Insured)	66	134	200	
None of the Above	18	21	39	
Have Heard About Osteoporosis:				
Yes	255	468	723	P≤ 0.013
No	18	13	31	
Have been diagnosed before or one of their relatives with osteoporosis.				
Yes	84	216	300	P≤ 0.000
No	189	265	454	

Knowledge assessment using the OKATamong age groups and gender

Out of 18 total scores of OKAT, 7.93 was the mean score among our sample size with minimum and maximum scores of 0 and 18, respectively, the correlation between those who had and had not heard about osteoporosis and the total score of the tool is significant P≤ 0.001. The relationship between total scores of OKAT and age groups was a significant P≤ 0.000, particularly between age groups range of 15 until 29to 30 until 44P≤ 0.031 and between age groups of 15 until 29to45 until 60 P≤ 0.001. Similarly, the relationship between gender and total scores of OKAT was significant P≤ 0.000. Other knowledge assessments among age groups and gender are shown in Tables 2, 3.

**Table 2:** Age groups and knowledge assessment using the Osteoporosis Knowledge Assessment Tool (OKAT) n = Total(Means ± Standard Deviation),P: Partial correlation coefficients P-value was carried out using bivariate statistics such as Chi-square and one-way analysis of variance (ANOVA).

Categories	Means ± Standard Deviation	Minimum Values	Maximum Values	P-values of the General Relationship
15 to 29 Years old. n= 467	(7.55±2.9)	0	18	P≤ 0.000
30 to 44 Years old. n=176	(8.33±3.0)	1	16	
45 to 60 Years old. n=105	(8.88±3.1)	2	16	
More than 60 Years old. n=6	(8.50±2.7)	5	12	
Total Sample Size n=754	(7.92±3.0)	0	18	

**Table 3:** Gender and knowledge assessment using the Osteoporosis Knowledge Assessment Tool (OKAT) (Means ± Standard Deviation),P: Partial correlation coefficients P-value was carried out using bivariate statistics such as Chi-square and one-way analysis of variance (ANOVA).

Categories	Total OKAT Score	Total	P-values
Males	(7.37±3.1)	273	P≤ 0.000
Females	(8.26±2.8)	481	

Assessment of attitudes and beliefs by OHBSaccording to age groups and gender

The scale measured seven domains; Susceptibility, Seriousness, Benefits Exercise, Benefits Calcium Intake, Barriers Exercise, Barriers Calcium Intake, and Health Motivation; each domain had a total score of 30 points, and the total score of the scale was 210 points. The values of the overall total scale ranged between 42 and 210. All total scale scores of domains and the overall total scale score were statistically significant with age groups P≤ 0.042 except for Benefits Exercise and Benefits Calcium, which had P≤ 0.940 and P≤ 0.505, respectively. Lastly, the overall total scale score of OHBS and age groups between 15 until 29and 45 until 60P≤ 0.000. The majority of the relationships between gender and OHBS domains were insignificant except for the domains of susceptibility, barriers exercise, and barriers calcium intake, which were statistically significant P≤ 0.000, P≤ 0.001, and P≤ 0.005, respectively. Full detailed information on the assessment of attitudes and knowledge of OHBS with to age groups refer to Tables 4, 5.

**Table 4:** Full details regarding the relationships between OHBS domains and age groups (Means ± Standard Deviation),P: Partial correlation coefficients P-value was carried out using bivariate statistics such as Chi-square and one-way analysis of variance (ANOVA).

Categories	Age Groups	Totals	Means ± Std. Deviations	Minimum Values	Maximum Values	P- Values of the General Relationship
Susceptibility	15 to 29	467	21.91±4.979	6	30	P≤ 0.000
	30 to 44	176	20.62±4.553	9	30	
	45 to 60	105	18.81±5.144	6	30	
	More than 60	6	21.33±3.882	17	28	
	Total	754	21.17±5.010	6	30	
Seriousness	15 to 29	467	19.60±5.140	6	30	P≤ 0.007
	30 to 44	176	19.06±4.929	6	30	
	45 to 60	105	17.74±4.682	6	29	
	More than 60	6	20.33 ±3.327	15	24	
	Total	754	19.22±5.050	6	30	
Benefits Exercise	15 to 29	467	13.80±6.376	6	30	P≤ 0.940
	30 to 44	176	13.66±6.375	6	30	
	45 to 60	105	13.53±6.019	6	30	
	More than 60	6	12.50±1.378	11	15	
	Total	754	13.72±6.296	6	30	
Benefits Calcium Intake	15 to 29	467	15.58±5.385	6	30	P≤ 0.505
	30 to 44	176	15.71±5.619	6	30	
	45 to 60	105	14.83±4.669	6	30	
	More than 60	6	14.33±2.422	10	17	
	Total	754	15.50±5.331	6	30	
Barriers Exercise	15 to 29	467	20.70±4.500	6	30	P≤ 0.420
	30 to 44	176	21.39±4.244	6	30	
	45 to 60	105	19.92±3.807	6	30	
	More than 60	6	22.17±4.355	16	29	
	Total	754	20.76±4.365	6	30	
Barriers Calcium Intake	15 to 29	467	18.38±4.080	6	30	P≤ 0.000
	30 to 44	176	17.43±3.823	6	29	
	45 to 60	105	16.58±3.527	6	27	
	More than 60	6	15.50±1.761	14	19	
	Total	754	17.89±3.990	6	30	
Health Motivation	15 to 29	467	18.89±4.323	6	30	P≤ 0.000
	30 to 44	176	18.31±3.658	7	28	
	45 to 60	105	17.25±3.658	6	28	
	More than 60	6	14.17±3.061	9	18	
	Total	754	18.49±4.130	6	30	
Total Scale Score of OHBS	15 to 29	467	128.86±22.741	42	210	P≤ 0.000
	30 to 44	176	126.18±22.064	46	197	
	45 to 60	105	118.67±19.917	42	186	
	More than 60	6	120.33±7.659	108	128	
	Total	754	126.74±22.375	42	210	

**Table 5:** Gender and domains of Osteoporosis Health Belief Scale (OHBS) n = Total(Means ± Standard Deviation),P: Partial correlation coefficients P-value was carried out using bivariate statistics such as Chi-square and one-way analysis of variance (ANOVA).

Domains of OHBS	Gender	Totals	(Means± Std. Deviation)	P-values of the Relationships
Susceptibility	Males	273	22.17±5.171	P≤ 0.000
	Females	481	20.60±4.830	
Seriousness	Males	273	19.32±5.482	P≤ 0.690
	Females	481	19.16±4.793	
Benefits Exercise	Males	273	13.98±6.640	P≤ 0.388
	Females	481	13.57±6.094	
Benefits Calcium Intake	Males	273	15.57±5.558	P≤ 0.782
	Females	481	15.46±5.204	
Barriers Exercise	Males	273	20.09±4.553	P≤ 0.002
	Females	481	21.15±4.211	
Barriers Calcium Intake	Males	273	18.42±4.290	P≤ 0.007
	Females	481	17.58±3.779	
Health Motivation	Males	273	18.65±4.508	P≤ 0.426
	Females	481	18.40±3.902	
Total Scale Score of OHBS	Males	273	128.20±24.779	P≤ 0.178
	Females	481	125.92±20.869	
.				

Knowledge assessment using the OKATand OHBSamong educational levels of our respondents

After we deployed one-way ANOVA tests, all of the statistical relationships on both OKAT and OHBS were insignificant, except for the domain of Barriers Exercise from OHBS which was significant P≤ 0.002, particularly between (Post Graduate or Doctoral) to both (Intermediate or High School)P≤ 0.002 and (Bachelor College Degree) P≤ 0.040. Refer to Tables 6, 7 for full details.

**Table 6:** Knowledge assessment using the Osteoporosis Knowledge Assessment Tool (OKAT) among educational levels of the respondents. n = Total(Means ± Standard Deviation),P: Partial correlation coefficients P-value was carried out using bivariate statistics such as Chi-square and one-way analysis of variance (ANOVA).

Categories	Means ± Standard Deviation	Minimum Values	Maximum Values	P-values of the General Relationship
Elementary or Less. n= 0	(0±0)	0	0	P≤ 0.000
Intermediate or High School. n=249	(7.74±2.9)	0	18	
College Degree (bachelor). n=105	(8.00±3.5)	0	16	
Post-graduate/Doctoral. n=6	(8.18±3.5)	0	16	
Total Sample Size n=754	(7.93±3.0)	0	18	

**Table 7:** Knowledge assessment Osteoporosis Health Belief Scale (OHBS) in accordance with educational levels n = Total(Means ± Standard Deviation),P: Partial correlation coefficients P-value was carried out using bivariate statistics such as Chi-square and one-way analysis of variance (ANOVA).

Categories		Totals	(Means ± Std. Deviations)	Minimum	Maximum	P-values of the General Relationship
Susceptibility	Elementary or Less	0	0±0	0	0	P≤0.410
	Intermediate or High Schools	249	21.51±5.102	6	30	
	College Degree (Bachelor)	431	21.02±4.990	6	30	
	Post Graduate/Doctoral	74	20.89±4.816	6	30	
	Total	754	21.17±5.010	6	30	
Seriousness	Elementary or Less	0	0±0	0	0	P≤0.723
	Intermediate or High Schools	249	19.16±5.326	6	30	
	College Degree (Bachelor)	431	19.32±4.874	6	30	
	Post Graduate/Doctoral	74	18.82±5.151	6	30	
	Total	754	19.22±5.050	6	30	
Benefits Exercise	Elementary or Less	0	0±0	0	0	P≤0.613
	Intermediate or High Schools	249	13.41±6.286	6	30	
	College Degree (Bachelor)	431	13.91±6.333	6	30	
	Post Graduate/Doctoral	74	13.65±6.152	6	30	
	Total	754	13.72±6.296	6	30	
Benefits Calcium Intake	Elementary or Less	0	0±0	0	0	P≤0.409
	Intermediate or High Schools	249	15.15±5.628	6	30	
	College Degree (Bachelor)	431	15.63±5.185	6	30	
	Post Graduate/Doctoral	74	15.92±5.152	6	30	
	Total	754	15.50±5.331	6	30	
Barriers Exercise	Elementary or Less	0	0±0	0	0	P≤0.002
	Intermediate or High Schools	249	20.19±4.414	6	30	
	College Degree (Bachelor)	431	20.84±4.294	6	30	
	Post Graduate/Doctoral	74	22.23±4.283	6	29	
	Total	754	20.76±4.365	6	30	
Barriers Calcium Intake	Elementary or Less	0	0±0	0	0	P≤0.167
	Intermediate or High Schools	249	18.07±4.178	6	30	
	College Degree (Bachelor)	431	17.92±3.900	6	30	
	Post Graduate/Doctoral	74	17.08±3.813	6	29	
	Total	754	17.89±3.990	6	30	
Health Motivation	Elementary or Less	0	0±0	0	0	P≤0.583
	Intermediate or High Schools	249	18.62±4.390	6	30	
	College Degree (Bachelor)	431	18.49±4.060	6	30	
	Post Graduate/Doctoral	74	18.05±3.630	6	28	
	Total	754	18.49±4.130	6	30	
Total Scale Score of OHBS	Elementary or Less	0	0±0	0	0	P≤0.854
	Intermediate or High Schools	249	126.12±23.933	42	210	
	College Degree (Bachelor)	431	127.12±21.398	42	206	
	Post Graduate/Doctoral	74	126.65±22.777	42	197	
	Total	754	126.74±22.375	42	210	

## Discussion

Osteoporosis has been associated with increased patient morbidity [15]. Although tons of health campaigns were made to target the public for the diseaseand to increase their knowledge, patient outcomes did not differ over the past years. Patients’ knowledge of their illness has shown to be one of the essential factors for better health outcomes [16]. Recent emerging evidence showed not only knowledge but beliefs could also be related. Health-related behaviors have been one of the determinant factors for better patient outcomes [6]. Therefore, to reach this level of awareness, the population at risk must acknowledge their susceptibility to the disease.

Consequently, they would take initiatives such as actions to prevent the occurrence of the disease, early screening,or even following a strict management plan [6]. Unfortunately, this is an uncharted territory that has not been addressed in our local community. To our knowledge and after a comprehensive literature review, it is evident that this is the first study in Saudi Arabia to examine osteoporosis knowledge using the OKAT questionnaire. Moreover, the study examined the public beliefs toward osteoporosis using the OHBS questionnaire that involved a wide range of age groups, including both genders.

Regarding the assessment of the knowledge, 95.9%of the study participants had heard about osteoporosis. This result is consistent with a study done in 2015, which included the main regions of Saudi Arabia [7]. Our findings are also in line with a study among El-Salvador women (75%) [17]. Likewise, 73%of the general Iranian population and 90%of Turkish women had heard about the disease [18,19]. However, our result is inconsistent with a study carried out in India, in which 70%of participants were not aware of the disease [20]. Such variants can be explained by the deficit of awareness among those individuals, which has been reported in a recent study done in India [21].

In terms of osteoporosis knowledge between genders, the score of female participants was higher than males. The difference between females and males may be attributed to the misunderstanding that osteoporosis only affects women. Furthermore, most health education and awareness campaigns focus on women in Saudi Arabia, which may lead women to acquire more knowledge about diseases they are at higher risk for. Similar results were reported in a study conducted in Riyadh by Barzanji et al. [22]. However, a study in Iran could not find such an association [18].

The level of education could affect the awareness of osteoporosis. It can influence behavior, personal health responsibility, the ability to acquire, and to understand health information [23]. In the present study, there was no significant statistical relationship between the education level of participants and the overall knowledge of osteoporosis. This result is consistent with a study done by Riaz et al. [11]. It reported no significant difference in the scores of highly educated and low-educated women in their study. However, our result is inconsistent with other studies that stated a positive relationship between the two.

Regarding age and assessment of knowledge toward osteoporosis, our study reported the younger the individual, the more knowledgeable he or she is. It is consistent with other studies where they stated similar results [7,24]. Although it has been reported that age can affect the level of knowledge, osteoporosis’s knowledge increases with age [25]. While a study was done on the general Iranian population, they could not find an association between age and awareness level [6]. Our results could be explained by the fact that young individuals have more access to the new wellsprings of information (i.e., internet and smartphones).

Our sample had a lower mean score of total OHBS, which reflects a serious problem regarding the beliefs towardosteoporosis. Moreover, our sample showed some areas of defects, mostly in the benefits of exercise and calcium intake. These two elements represent the major factors for underlying mechanisms to develop osteoporosis [26]. Furthermore, our sample had a lower total score of OHBS compared to the Korean study [26]. Nevertheless, we shared similar scores for the benefit of exercise and calcium intake, which explains global misunderstanding, rather than a local variance, when tackling this area of conflict.

On the other hand, our sample scored relatively higher in the categories of susceptibility and seriousness compared to the study, as mentioned earlier [26]. Both of these had a statistically significant relationship, which can implicate the relative knowledge that our sample has of the Koreans. However, this variance might be attributed to the difference in the nature of our sample, which involved male participants, unlike the Korean study.

Elderly individuals are more prone to develop osteoporosis than the rest of the population, making them perceive themselves as a higher-risk group [6]. Besides, the results showed that they had scored higher in the categories of susceptibility and seriousness compared to other age groups. Likewise, our sample had a higher score than the studies mentioned above for this age group. This result comes as no surprise, as health-based model behaviors suggested that younger individuals might perceive themselves as not susceptible to this disease, therefore they might score lower than the older age group [27]. Surprisingly, the results demonstrated that younger age groups had higher scores in the category of susceptibility than the older age groups. This result is inconsistent with the previous literature.

Although 27% of men older than 50 years of age have been diagnosed with osteoporosis [28], the public still perceives osteoporosis as a woman’s health issue. Such behaviors would not make them take preventive measures to avoid it as calcium intake and physical activities. Notably, the results showed that male participants had significantly higher scores in most of the categories.

Limitations

Due to the nature of this cross-sectional study, a generalization of our findings and causation cannot be made. Moreover, this is the first local study to examine osteoporosis using validated translated OKAT and OHBS questionnaires with a broader range of participants. It did not allow us to compare our results with other local communities within our kingdom. Furthermore, the population at hand is most likely missing the low socioeconomic status communities due to limited access to online social platforms and, consequently, less awareness of osteoporosis.

## Conclusions

Although our sample had higher scores in knowledge in regard to previous literature, their belief scores were lower. It raises the notion to target various aspects of belief perception in well-designed, cultural-appropriate, tailored awareness programs to be implemented, that address all age and gender groups. These programs should focus on education materials regarding osteoporosis, healthy eating habits, and physical activities. Moreover, we recommend other researchers to conduct a population-based cross-sectional study to address the knowledge and beliefs related to the participants’ dual-energy X-ray absorptiometry (DXA) scan findings.

## Appendices

**Table 8:** The Osteoporosis Knowledge Assessment Tool (OKAT)

Please answer each of the following questions with True, False or Don’t Know.			
1. Osteoporosis leads to an increased risk of bone fractures.	True	False	Don’t know
2. Osteoporosis usually causes symptoms ( e.g. pain) before fractures occur.	True	False	Don’t know
3. Having a higher peak bone mass at the end of childhood gives no protection against the development of osteoporosis in later life.	True	False	Don’t know
4. Osteoporosis is more common in men.	True	False	Don’t know
5. Cigarette smoking can contribute to osteoporosis.	True	False	Don’t know
6. White women are at highest risk of fracture as compared to other races.	True	False	Don’t know
7. A fall is just as important as low bone strength in causing fractures.	True	False	Don’t know
8. By age 80, the majority of women have osteoporosis.	True	False	Don’t know
9. From age 50, most women can expect at least one fracture before they die.	True	False	Don’t know
10. Any type of physical activity is beneficial for osteoporosis.	True	False	Don’t know
11. It is easy to tell whether I am at risk of osteoporosis by my clinical risk factors.	True	False	Don’t know
12. Family history of osteoporosis strongly predisposes a person to osteoporosis.	True	False	Don’t know
13. An adequate calcium intake can be achieved from two glasses of milk a day.	True	False	Don’t know
14. Sardines and broccoli are good sources of calcium for people who cannot take dairy products.	True	False	Don’t know
15. Calcium supplements alone can prevent bone loss.	True	False	Don’t know
16. Alcohol in moderation has little effect on osteoporosis.	True	False	Don’t know
17. A high salt intake is a risk factor for osteoporosis.	True	False	Don’t know
18. There is a small amount of bone loss in the ten years following the onset of menopause.	True	False	Don’t know
19. Hormone therapy prevents further bone loss at any age after menopause.	True	False	Don’t know
20. There are no effective treatments for osteoporosis available in Saudi Arabia.	True	False	Don’t know

**Table 9:** Arabic translation of the Osteoporosis Knowledge Assessment Tool (OKAT)

لا اعرف	خطآ	صح	السؤال:
			١- هشاشة العظام تؤدي الي زيادة خطر الإصابة بكسر العظام
			٢-هشاشة العظام أحيانا تسبب اعراض مثل "الألم" قبل حدوث الكسر
			٣-وجود اعلى كتلة عظام في نهاية سن الطفولة لا يوفر حماية من إصابتك من هشاشة العظام في وقت لاحق من الحياة
			٤-هشاشة العظام أكثر شيوعا في الرجال
			٥- تدخين السجائر يمكن أن يساهم في هشاشة العظام
			٦-النساء ذوات البشرة البيضاء هم في أعلى خطورة للكسور مقارنة بغيرهن
			٧- السقوط لا يقل أهمية عن انخفاض قوة العظام في التسبب في الكسور
			٨- بحلول سن 80 عاما، فإن غالبية النساء لديهن هشاشة العظام
			٩- من سن 50، معظم النساء يمكن أن يتوقعن كسر واحد على الأقل قبل أن يتوفين.
			١٠- أي نوع من النشاط البدني مفيد لهشاشة العظام.
			١١- من السهل معرفة ما اذا كنت على خطر الإصابة بهشاشة العظام عن طريق فحوصاتي الإكلينيكية
			١٢- التاريخ العائلي بمرض هشاشة العظام يزيد من احتمالية إصابة الشخص بالمرض
			١٣- كأسين من الحليب يومي ا يساعد على أخذ الكمية الكافية من الكالسيوم
			١٤- الساردين والبروكلي من المصادر الجيدة للكالسيوم للأشخاص الذين لا يستطيعون أكل منتجات الألبان
			١٥- منتجات الكالسيوم لوحدها تستطيع منع مرض فقدان العظام
			١٦- الاستخدام المعتدل للكحول لديه تأثير على مرض هشاشة العظام
			١٧- اكل كمية كبيرة من الملح من العوامل المؤدية للهشاشة العظام
			١٨-هنالك كمية بسيطة من فقدان العظام في العشر السنين التالية لسن اليأس
			١٩- العلاج الهرموني يمنع المزيد من فقدان العظام بعد أي سن من بداية سن اليأس
			٢٠- لا توجد أي علاجات فعالة للهشاشة العظام متوفرة في السعودية

**Table 10:** Osteoporosis Health Belief Scale (OHBS)

	Strongly Disagree	Disagree	Neutral	Agree	Strongly Agree
1. Your chances of getting osteoporosis are high...............................................					
2. Because of your body build, you are more likely to develop osteoporosis...					
3. It is extremely likely that you will get osteoporosis........................................					
4. There is a good chance that you will get osteoporosis..................................					
5. You are more likely than the average person to get osteoporosis..................					
6. Your family history makes it more likely that you will get osteoporosis...					
7. The thought of having osteoporosis scares you...........................................					
8. If you had osteoporosis you would be crippled...............................................					
9. Your feelings about yourself would change if you got osteoporosis...........					
10. It would be very costly if you got					
11. When you think about osteoporosis you get depressed................................					
12. It would be very serious if you got osteoporosis........................................					
	Strongly Disagree	Disagree	Neutral	Agree	Strongly Agree
13. Regular exercise prevents problems that would happen from osteoporosis.					
14. You feel better when you exercise to prevent osteoporosis...........................					
15. Regular exercise helps to build strong bones...................................................					
16. Exercising to prevent osteoporosis also improves the way your body					
17. Regular exercise cuts down the chances of broken bones.....................					
18. You feel good about yourself when you exercise to prevent osteoporosis…….					
For the following 6 questions (numbers 19 to 24), "taking in enough calcium" means taking enough calcium by eating calcium rich foods and/or taking calcium supplements					
19. Taking in enough calcium prevents problems from osteoporosis...............					
20. You have lots to gain from taking in enough calcium to prevent osteoporosis........................................					
21. Taking in enough calcium prevents painful osteoporosis............................					
22. You would not worry as much about osteoporosis if you took in enough calcium...............................................					
23. Taking in enough calcium cuts down on your chances of broken bones.......					
24. You feel good about yourself when you take in enough calcium to prevent osteoporosis............................. ………….					
25. You feel like you are not strong enough to exercise regularly...............					
26. You have no place where you can exercise...............................................					

**Table 11:** Arabic translation of the Osteoporosis Health Belief Scale (OHBS)

البيان	لا أوافق بشدة	لا أوافق	محايد	أوافق	أوافق بشدة
١- فرص حصولك على هشاشة العظام مرتفعة					
٢- بسبب بٌنيتك الجسمية، أنت أكثر عرضة للإصابة بهشاشة العظام					
٣- من المرجح للغاية أنك سوف تصاب بهشاشة العظام					
٤- هناك فرصة جيدة أنك سوف تصاب بهشاشة العظام					
٥- أنت أكثر عرضة من الشخص العادي للإصابة بهشاشة العظام					
٦- تاريخك العائلي يجعلك من المرجح أن تصاب بـ هشاشة العظام					
٧- فكرة الإصابة بهشاشة العظام تخيفك					
٨- إذا كان لديك هشاشة العظام سوف تكون مقعد					
٩- سوف تتغير مشاعرك تجاه نفسك إذا كنت تعاني من هشاشة العظام					
١٠- سيكون مكلفا جدا إذا أُصبت بهشاشة العظام					
١١- عندما تفكر في هشاشة العظام تصبح مكتئب					
١٢- سيكون أمرا جديا جدا إذا اصبت بهشاشة العظام					
١٣- التمارين المنتظمة تمنع المشاكل التي قد تحدث من هشاشة العظام					
١٤- ستشعر أنك أفضل عند ممارسة الرياضة للوقاية من هشاشة العظام					
١٥-التمارين المنتظمة تساعد على بناء عظام قوية					
١٦-التمرن للوقاية من هشاشة العظام أيضا يحسن من المظهر الخارجي لجسمك					
١٧- ممارسة التمارين الرياضية بانتظام تقلل من فرص كسر العظام					
١٨- ستشعر بالرضا عن نفسك عندما تقوم بممارسة الرياضة للوقاية من هشاشة العظام					
من سؤال ١٩-٢٤ "اكل كمية كافية من الكالسيوم" تعني أخذها من مصادرها الغذائية أو من مكملات غذائية					
١٩- اكل كمية كافية من الكالسيوم يمنع مشاكل هشاشة العظام					
٢٠- لديك الكثير لكسبه من اكل كمية كافية من الكالسيوم لمنع هشاشة العظام					
٢١- اكل كمية كافية من الكالسيوم يمنع آلام هشاشة العظام					
٢٢- أنت لن تقلق كثيرا عن هشاشة العظام إذا كنت تأكل كمية كافية من الكالسيوم					
٢٣- اكل كمية كافية من الكالسيوم يقلل من عرضتك لفقدان العظام					
٢٤- تشعر برضا عن نفسك عندما تأخذ احتياجك الكافي من الكالسيوم لمنع الإصابة بهشاشة الامراض					
٢٥- تشعر بأنك لست قوي للدرجة التي تؤهلك لعمل التمارين بانتظام					
٢٦- لا تمتلك مكان للتدرب					
٢٧- شريك حياتك أو عائلتك تثبط من عزيمتك تجاه التمارين					
٢٨- التدرب بانتظام تعني البدء بهواية جديدة صعب القيام بها					
٢٩- التدرب بانتظام يجعلك مرهق					
٣٠- التدرب بانتظام يفسد روتينك اليومي					
٣١- الأغذية الغنية بالكالسيوم مكلفة					
٣٢- الأغذية الغنية بالكالسيوم لا تتوافق معك					
٣٣- لا تفضل الأغذية الغنية بالكالسيوم					
٣٤- أكل الأغذية الغنية بالكالسيوم يعني تغيير نظام الغذائي والذي هو صعب الحصول					
٣٥- لأجل اكل أغذية تحتوي على الكالسيوم أكثر عليك التوقف عن الأطعمة المفضلة لديك					
٣٦- الأغذية الغنية بالكالسيوم تحتوي على الكوليسترول					
٣٧- أنت تأكل وجبة غذائية متوازنة					
٣٨- أنت تبحث عن معلومات جديدة مرتبطة بالصحة					
٣٩- من المهم بالنسبة إليك البقاء صحياً					
٤٠-أنت تحاول على الكشف على الأخطاء الصحية مبكراً					
٤١- أنت تقوم بالفحوصات الصحية بانتظام حتى في حالة عدم المرض					
٤٢- أنت تبحث عن توصيات لتبقيك صحياً					

## References

[1] Ross HA, Lentjes E, Menheere PP (2011). The consensus statement on the standardization and evaluation of growth hormone and insulin-like growth factor assays lacks a recommendation to attempt efficacious harmonization. Clin Chem.

[2] Reginster JY, Burlet N (2006). Osteoporosis: a still increasing prevalence. Bone.

[3] Al-Saleh Y, Sulimani R, Sabico S (2015). 2015 guidelines for osteoporosis in Saudi Arabia: recommendations from the Saudi Osteoporosis Society. Ann Saudi Med.

[4] Cosman F, de Beur SJ, LeBoff MS, Lewiecki EM, Tanner B, Randall S, Lindsay R (2014). Clinician’s Guide to prevention and treatment of osteoporosis. Osteoporos Int.

[5] (2014). National Osteoporosis Guideline Group. Guideline for the diagnosis and management of osteoporosis. Executive summary.

[6] Shanthi Johnson C, McLeod W, Kennedy L, McLeod K (2008). Osteoporosis health beliefs among younger and older men and women. Health Educ Behav.

[7] Alamri FA, Saeedi MY, Mohamed A, Barzanii A, Aldayel M, Ibrahim AK (2015). Knowledge, attitude, and practice of osteoporosis among Saudis: a community-based study. J Egypt Public Health Assoc.

[8] Swaim RA, Barner JC, Brown CM (2008). The relationship of calcium intake and exercise to osteoporosis health beliefs in postmenopausal women. Res Social Adm Pharm.

[9] Winzenberg TM, Oldenburg B, Frendin S, Jones G (2003). The design of a valid and reliable questionnaire to measure osteoporosis knowledge in women: the Osteoporosis Knowledge Assessment Tool (OKAT). BMC Musculoskelet Disord.

[10] Kasper MJ, Garber M, Walsdorf K (2007). Young women’s knowledge and beliefs about osteoporosis: results from a cross-sectional survey of college females. Am J Heal Educ.

[11] Riaz M, Abid N, Patel J, Tariq M, Khan MS, Zuberi L (2008). Knowledge about osteoporosis among healthy women attending a tertiary care hospital. J Pak Med Assoc.

[12] UNFCCC. Saudi Arabia. UNFCCC Process - Parties. https://unfccc.int/node/61163.

[13] Whatsapp Messenger. https://web.whatsapp.com/.

[14] Twitter. https://twitter.com/home.

[15] NIH Consensus Development Panel on Osteoporosis Prevention, Diagnosis Diagnosis, and Therapy (2001). Osteoporosis prevention, diagnosis, and therapy. JAMA.

[16] von Hurst PR, Wham CA (2007). Attitudes and knowledge about osteoporosis risk prevention: a survey of New Zealand women. Public Health Nutr.

[17] Stolt M, Suhonen R, Puukka P, Viitanen M, Voutilainen P, Leino-Kilpi H (2015). Nurses' knowledge of foot care in the context of home care: a cross-sectional correlational survey study. J Clin Nurs.

[18] Hernandez-Rauda R, Martinez-Garcia S (2004). Osteoporosis-related life habits and knowledge about osteoporosis among women in El Salvador: a cross-sectional study. BMC Musculoskelet Disord.

[19] Amani F, Ghorbani A, Ghezelbash S, Barak M, Frazaneh E (2015). The level of people’s awareness of osteoporosis in Ardabil city: a survey based study. Int J Med Res Heal Sci.

[20] Ungan M, Tümer M (2001). Turkish women's knowledge of osteoporosis. Fam Pract.

[21] Jagarlamudi M, Rao V, Nitya K, Prathima P, Rana S (2015). A community based study on knowledge, attitudes and practice of osteoporosis in women. Indo Am J Pharm Res.

[22] Gopinathan NR, Sen RK, Behera P, Aggarwal S, Khandelwal N, Sen M (2016). Awareness of osteoporosis in postmenopausal Indian women: an evaluation of Osteoporosis Health Belief Scale. J Midlife Health.

[23] Barzanji AT, Alamri FA, Mohamed AG (2013). Osteoporosis: a study of knowledge, attitude and practice among adults in Riyadh, Saudi Arabia. J Community Health.

[24] Al Attia HM, Abu Merhi AA, Al Farhan MM (2008). How much do the Arab females know about osteoporosis? The scope and the sources of knowledge. Clin Rheumatol.

[25] Akinpetide GO (2014). Osteoporosis knowledge, beliefs, and bone promotion behaviors of postmenopausal African American (AA) women. https://www.semanticscholar.org/paper/Osteoporosis-Knowledge%2C-Beliefs%2C-and-Bone-Promotion-Akinpetide/c0148120537b0ce9d6bd0909de24bed9f7c2c74b#citing-papers.

[26] Kim TH, Lee YS, Byun DW, Jang S, Jeon DS, Lee HH (2013). Evaluation of the osteoporosis health belief scale in korean women. J Bone Metab.

[27] Glanz K (1997). Theory at a glance: A guide for health promotion practice. National Cancer Institute.

[28] Cooley H, Jones G (2001). A population-based study of fracture incidence in southern Tasmania: lifetime fracture risk and evidence for geographic variations within the same country. Osteoporos Int.

